# Supplementary material for: Arterial floating mural thrombi are a characteristic imaging pattern in SARS-CoV-2-related ischemic stroke
Source: PLoS One. 2024 Oct 25;19(10):e0311622. doi: 10.1371/journal.pone.0311622 (PMC11508162; doi:10.1371/journal.pone.0311622)
Supplement: S1 Table — (DOCX) [file pone.0311622.s001.docx]

| **S1 Table** | | | |
| --- | --- | --- | --- |
| Vascular anatomy for LVO patients | | | |
|  | **SARS-CoV-2 n=18** | **non- SARS-CoV-2 n=17** | p-value |
| M1 | 3 (8.6) | 4 (11.8) | 0.708 |
| M2 | 2 (5.7) | 3 (8.8) |  |
| M3 | 2 (5.7) | 0 (0.0) |  |
| PCA | 4 (11.4) | 2 (5.9) |  |
| ACA | 1 (2.9) | 1 (2.9) |  |
| T | 2 (5.7) | 1 (2.9) |  |
| Vert | 1 (2.9) | 1 (2.9) |  |
| Tandem | 2 (5.7) | 1 (2.9) |  |
| Carotid | 1 (2.9) | 4 (11.8) |  |
|  | | | |
