# Supplementary material for: Arterial floating mural thrombi are a characteristic imaging pattern in SARS-CoV-2-related ischemic stroke
Source: PLoS One. 2024 Oct 25;19(10):e0311622. doi: 10.1371/journal.pone.0311622 (PMC11508162; doi:10.1371/journal.pone.0311622)
Supplement: S2 Table — (DOCX) [file pone.0311622.s002.docx]

| **S2 Table** | | | |
| --- | --- | --- | --- |
| Stroke outcomes | | | |
|  | **SARS-CoV-2** | **control** | p-value |
| Functional independence at day90 (mRS=0-2), n(%) | 13 (41.9) | 17 (53.1) | 0.374 |
| mRS, median (IQR) | 3 (1-6) | 2 (1-4) | 0.537 |
| Mortality, n (%) | 11 (31.4) | 6 (17.1) | 0.163 |
|  | | | |
