# Supplementary material for: Arterial floating mural thrombi are a characteristic imaging pattern in SARS-CoV-2-related ischemic stroke
Source: PLoS One. 2024 Oct 25;19(10):e0311622. doi: 10.1371/journal.pone.0311622 (PMC11508162; doi:10.1371/journal.pone.0311622)
Supplement: S3 Table — (DOCX) [file pone.0311622.s003.docx]

| **S3 Table** | | | | |
| --- | --- | --- | --- | --- |
| Predictors of higher mRS score | | | | |
|  |  | **95% C.I.** | |  |
|  | **OR** | **Lower** | **Upper** | p-value |
| Age | 1.061 | 1.011 | 1.114 | **0.017** |
| CRP | 1.006 | 0.898 | 1.126 | 0.924 |
| SARS-CoV-2 related stroke | 1.675 | 0.567 | 4.944 | 0.35 |
| IVT | 0.639 | 0.167 | 2.437 | 0.512 |
| EVT | 0.64 | 0.176 | 2.335 | 0.499 |
| Multivariant ordinal regression within entire cohort | | | | |
