# Supplementary material for: Arterial floating mural thrombi are a characteristic imaging pattern in SARS-CoV-2-related ischemic stroke
Source: PLoS One. 2024 Oct 25;19(10):e0311622. doi: 10.1371/journal.pone.0311622 (PMC11508162; doi:10.1371/journal.pone.0311622)
Supplement: S4 Table — (DOCX) [file pone.0311622.s004.docx]

| **S4 Table** | | | |
| --- | --- | --- | --- |
| Sub-analysis of SARS-CoV-2 patient’s characteristics | | | |
|  | **Floating thrombus n =5** | **No floating thrombus n = 30** | P - value |
| **Demographic and clinical characteristics** |  | | |
| Age, median (IQR) | 78 (72.5,80) | 73.5 (64,81.75) | 0.185 |
| Female, n (%) | 1 (20) | 13 (43.3) | 0.627 |
| HTN, n (%) | 4 (80) | 20 (66.7) | 0.491 |
| AF, n (%) | 0 (0) | 9 (30) | 0.203 |
| DM, n (%) | 4 (80) | 18 (60) | 0.374 |
| Dyslipidemia, n (%) | 1 (20) | 18 (60) | 0.120 |
| Smoking, n (%) | 0 (0) | 7 (23.3) | 0.303 |
| IHD, n (%) | 0 (0) | 9 (30) | 0.203 |
| prior TIA/stroke, n (%) | 2 (40) | 8 (26.7) | 0.447 |
| **Time interval and stroke outcomes** |  | | |
| Time from COVID to stroke (days), median (IQR) | 7 (0,15) | 0 (0,8) | 0.945 |
| Time from COVID to stroke (days), mean (SD) | 7.4 (7.64) | 3.3 (6.71) | 0.230 |
| good functional outcome (mRS≤2), n (%) | 2 (40) | 11 (4.7) | 0.566 |
| mortality, n (%) | 1 (20) | 10 (33.3) | 0.491 |
| HTN, hypertension; AF, atrial fibrillation; IHD, ischemic heart disease; TIA, transient ischemic attack | | | |
